# Two patients walk into a clinic...a genomics perspective on the future of schizophrenia

**DOI:** 10.1186/1741-7007-9-77

**Published:** 2011-11-11

**Authors:** Aiden P Corvin

**Affiliations:** 1 Neuropsychiatric Genetics Research Group, Institute of Molecular Medicine and Department of Psychiatry, Trinity College Dublin, Dublin 2, Ireland

## Abstract

Progress is being made in schizophrenia genomics, suggesting that this complex brain disorder involves rare, moderate to high-risk mutations and the cumulative impact of small genetic effects, coupled with environmental factors. The genetic heterogeneity underlying schizophrenia and the overlap with other neurodevelopmental disorders suggest that it will not continue to be viewed as a single disease. This has radical implications for clinical practice, as diagnosis and treatment will be guided by molecular etiology rather than clinical diagnostic criteria.

## Opinion

In 2011, two people meet in a psychiatrist's waiting room. To the untrained eye they have little in common. Patient A has a history of brief psychotic episodes characterized by persecutory delusions and auditory hallucinations. She is married, with a good job and has been asymptomatic since starting medication three years before. Patient B is slow and unreactive in his responses, communicates poorly, has poor hygiene and is suspicious of other people. He believes that aliens have implanted a device in his head that controls his thoughts, feelings and actions. He hears them talking about him and commenting on his behavior. He has spent much of the last three years in hospital and has few remaining social contacts. Despite the obvious differences in symptomatology and illness course, their treating psychiatrist has diagnosed them both with schizophrenia, prescribes them the same medication and enrolls them as participants in a research study of schizophrenia.

**Figure 1:**
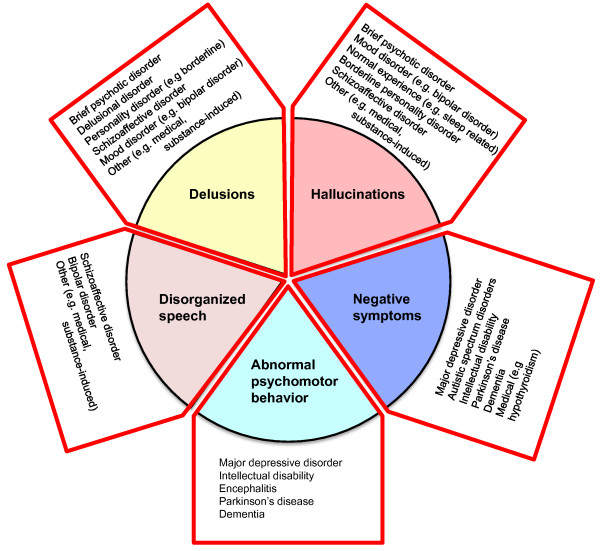
**Indicating the five main symptom domains in schizophrenia**. For a diagnosis, symptoms of these generally need to co-occur for one month or more. The figure shows how each of these symptom domains overlaps with other disorders.

Schizophrenia affects approximately 1% of the adult population and reduces life expectancy by an average of 20 to 25 years through the impact of the disorder on self-care and physical health, as well as through suicide [1]. At the present time the etiological mechanisms underlying schizophrenia are poorly understood. Schizophrenia is diagnosed clinically, based on characteristic symptoms of psychosis, disorganization and so called 'negative' symptoms (representing a reduced range of emotional expression, reduced production of speech and a lack of volition/motivation); duration of illness; impaired functioning; and the exclusion of other disorders such as autism and bipolar disorder. For clinicians, identifying which psychotic patients have schizophrenia requires clinical acumen and familiarity with the DSM-IV or ICD-10 diagnostic manuals [2,3]. Psychiatrists generally agree on cases where these criteria are met and this has helped standardize approaches to research and treatment, but as the vignette highlights, the symptoms are heterogeneous and outcome is variable even with treatment. Although the diagnostic criteria are stringent, at the level of individual symptoms schizophrenia overlaps with other psychiatric disorders, medical disorders and even with normal human experience (Figure 1).

Revisions of the criteria for clinical diagnosis have attempted to address such concerns, but the decision making process that underlies this process revolves around criteria that are unlikely to reflect underlying biological mechanisms. For example, in DSM-IV the requirement for a six-month period of continuous illness provides a clear distinction from brief psychotic disorders, but differs from the one-month criteria specified in ICD-10. In DSM-IV at least two characteristic symptoms must generally be present, namely: delusions, hallucinations, disorganized speech, grossly abnormal psychomotor behavior and negative symptoms. Only one symptom is required for specific types of auditory hallucinations (such as afflict patient B) or for delusions if they are bizarre. Tellingly, this is likely to change in future diagnostic guidelines (for example, in DSM-V due in 2013) as such features are not pathognomonic; that is, they are also reported in other psychotic conditions. In fact, none of the five characteristic symptoms are diagnosis-specific and to satisfy the criteria for schizophrenia other disorders (for example, psychosis due to a general medical condition) must first be excluded. Explicit in the criteria, the negative impact of the illness on social and occupational function is part of the diagnosis, although this will reflect a myriad of personal, family, cultural, societal and medical factors. Attempts to differentiate patients who meet the diagnostic criteria, such as A and B, based on clinical subtypes such as 'paranoid type' or 'disorganized type' have not proved fruitful either, as features of the illness can change over time. Indeed, these subtypes are likely to be abandoned in future guidelines.

Given such a challenging range of clinical phenotypes, it is not altogether surprising that animal models of schizophrenia based on diverse human symptoms, or resulting from serendipitous clinical observation, have yielded few insights and no new therapies [4]. Implicit in most models is the assumption that schizophrenia is a single disorder, but an equally plausible view, echoing Bleuler's earlier conceptualization of a 'group of schizophrenias' [5], is that the clinical phenotype may capture several or more distinct molecular pathologies or diseases. However, despite the fact that schizophrenia is challenging, it is also a substantially heritable phenotype. Taking this as a starting point, recent progress in genomics may be helpful in guiding researchers towards a better understanding of the biological origins of this disease or group of diseases.

## From genetic epidemiology to schizophrenia genes

**Figure 2:**
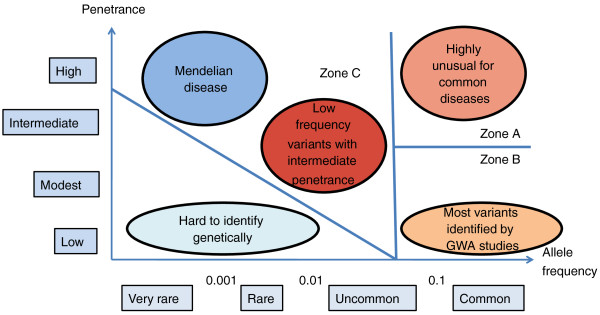
**Models of complex genetic etiology**. Risk variants are grouped according to their frequency in the population (x-axis) and their penetrance (y-axis). The penetrance of a disease-causing mutation is the proportion of individuals with the mutation who exhibit clinical symptoms. Zones A and B indicate risk variation assayed in genome-wide association studies (GWAS), and zone C indicates variants assayed by studies of copy number variation (CNV). Zone C also includes rare variants of intermediate penetrance that will be accessible to exomic and genomic sequencing (adapted from McCarthy *et al*. [36]).

From epidemiological studies of risk in schizophrenia patients and their relatives it has been suggested that several (or more) susceptibility genes interact with each other and with environmental risk to cause illness. This is consistent with the common disease common variant (CDCV) model, which proposes that multiple common alleles each make a small contribution to susceptibility, and may combine, together with environmental risk factors, to cause disease when a certain threshold is reached. An alternative view is that susceptibility involves the influence of rare genetic variants either contributing to a common disease or capturing multiple rare diseases. Only relatively recently have the required molecular research tools become available to begin empirically testing these hypotheses (Figure 2). Both common risk variants, such as single nucleotide polymorphisms (SNPs) with a frequency of greater than 5% in the population, but with individually small effects (odds ratio < 1.2) and rare variants with larger effects (odds ratio = 1.5 to ≥ 20) have been identified, provoking much speculation about the relative contribution of different classes or mechanisms of genetic risk and their potential interaction. And this is speculation, as collectively these risk variants at present explain only a modest proportion of total schizophrenia heritability (< 5%).

## Lessons learned from genome-wide association studies

Genome-wide association studies (GWAS) support the involvement of common risk variants and implicate a number of specific genetic risk variants. Using a novel polygene score method, the International Schizophrenia Consortium identified substantial overlap in common putative risk alleles of small effect across the genome in schizophrenia datasets and estimated that these explain at least one-third of total variation in schizophrenia liability [6]. The same set of multiple genetic variants of small effect were also associated in a bipolar disorder sample, supporting findings for overlapping risk between these disorders from family-based epidemiological studies [7]. This polygenic model has received support in a recent large meta-analysis of GWAS conducted by the Psychiatric GWAS Consortium (PGC) [8] and is informative in suggesting that hundreds of common risk variants may be involved in susceptibility without confirming which SNPs are contributory.

As of autumn 2011, a number of reported schizophrenia GWAS and a meta-analysis by the PGC have provided significant evidence for ten susceptibility loci [8-13] (Table 1). In keeping with the epidemiological data and polygene score analysis, many of these loci appear to confer liability to both schizophrenia and bipolar disorder [8]. Trying to move from these loci, many of unknown function, to a coherent molecular framework for schizophrenia is a daunting task, although one also faced in other common diseases, including diabetes and inflammatory bowel disease. Individually the variants are of modest effect (for example, increasing risk from 1 to 1.15) and collectively they account for a small proportion of total variance in risk; findings that are of limited diagnostic or prognostic utility. For biologists, investigating such small and probably subtle effects in model systems is challenging and may not be particularly informative. However, the ultimate goal of GWAS is the discovery of biological risk pathways underlying complex traits and there is little reason to believe that schizophrenia will prove different to other traits where progress has been made, including height and blood pressure [14,15].

**Table 1:** The main replicated risk variants identified for schizophrenia with their locations and effect sizes

Confirmed common risk variants for schizophrenia						
Chomosome	Variant	P-value	Odds Ratio	95% CI	Gene	Reference
1p21.3	rs1625579	1.5 × 10^-11^	1.12	1.09-1.16	*MIR 137*	[8]
2p15.1	rs2312147	1.9 × 10^-9^	1.09		*VRK2*	[13]
2q32.1	rs1344706	2.5 × 10^-11^	1.1	1.07-1.14	*ZNF804A*	[9]
2q32.3	rs17662626	4.65 × 10^-8^	1.2	1.13-1.26		[8]
6p21.3-p22.1*	rs2021722	2.18 × 10^-12^	1.15	1.11-1.19	*HLA *region	[8,10-12]
8p23.2	rs10503253	1.45 × 10^-8^	1.16	1.11-1.21	*CSMD1*	[8]
8q21.3	rs7004633	2.75 × 10^-8^	1.1	1.07-1.14		[8]
10q24.32*	rs7914558	2.23 × 10^-8^	1.22	1.15-1.29	*CNNM2*	[8]
11q24.2	rs12807809	2.8 × 10^-9^	1.16	1.09-1.24	*NRGN*	[8,11]
18q21.2*	rs12966547	2.35 × 10^-8^	1.4	1.28-1.52	*TCF4*	[8,10-12]
**Confirmed rare variant risks for schizophrenia**						
**Chomosome CNV type**	**Position (Mb)**	**P-value**	**Odds Ratio**	**95% CI**	**Gene**	**Reference**
1q21.1 del	143.8-146.6	2.2 × 10^-8^	8.3	3.7-19.9		[21-23]
1q21.1 dup	143.8-146.6	2 × 10^-3^	3.7	1.5-8.7		[23]
2p16.3 del	50.7-51.3	5.5 × 10^-9^	8.2	3.8-19.4	*NRXN1*	[23,25]
3q29 del	197.2-198.83	4 × 10^-4^	2.9			[23,24]
7q36.3 dup	158.7-158.81	8.3 × 10^-5^	16.4	3.11, infinity	*VIPR2*	[23,24]
15q11.2 del	20.3-20.8	6 × 10^-4^	2.73	1.5-4.89		[22]
15q13.3 del	28.2-30.6	2 × 10^-9^	9.9	4.3-24.4		[21-23]
16 dup	9.09-9.12	1 × 10^-4^	12.9	2.8-121.4	*C16orf72*	[23]
16p11.2 dup	15.0-18.0	1.5 × 10^-12^	11.6	5.6-29.3		[23]
16p13.1 dup	29.5-30.2	7 × 10^-3^	3.27	1.29-7.94		[26]
17p12 del	14.0-15.4	5 × 10^-5^	10	not presented		[27]
22q11 del	17.1-19.9	< 1.0 × 10^-16^	44	35.9-infinity		[23]

The Odds Ratio (OR) is a measure of effect size. It is the ratio of the odds of the variant occuring in the group of people with disease versus the ratio in the control group.

The 95% confidence interval (CI) gives the range within which the true OR lies with a 95% probability.

CNV denotes a copy number variant, which may either be a deletion (del) or duplication (dup).

An asterisk indicates that more than one variant has been implicated at this locus. Details for rare variants are provided where only one gene is implicated; typically, the other CNVs implicate 10 to 20 or more genes.

From the list of identified common schizophrenia risk loci, at least two (*MIR137 *and *ZNF804A*) appear to have a role in regulating other genes. Relatively little is known about the role of the microRNA 137 (*MIR137*) gene in brain function, although it has been implicated in neuronal maturation and adult neurogenesis. Interestingly, four of the other GWAS-implicated susceptibility genes (*TCF4*, *CACNA1C*, *CSMD1 *and *C10orf26*) have predicted miR-137 target sites. In the PGC dataset, SNPs mapping to the 301 high-confidence predicted gene targets of miR-137 were also enriched for association signals, compared with other genes of similar size or genetic marker density, making the *MIR13 *locus and network an attractive target for further investigation [8].

Larger GWAS datasets are being collected and may be useful in identifying additional common risk variants and further informing biology, a brute force approach that has been useful with other complex traits as described above [14,15]. Having a better estimation of small genetic effects can be informative for several reasons. At a summary level, it will be useful to have better estimates of the proportion of schizophrenia heritability captured by common SNPs and to know how this overlaps with other disorders. In the International Schizophrenia Consortium paper, one noteworthy finding from the polygene score analysis was that the common risk identified extended to bipolar disorder but not to seven other common medical disorders, including multiple sclerosis, diabetes and hypertension [6]. Analyses currently underway are assessing whether schizophrenia genetic risk extends across psychiatric phenotypes, including autism, depression and attention-deficit hyperactivity disorder (ADHD). This will be important in helping to determine whether these are etiologically related or distinct conditions.

Having better estimates of small effects may also clarify whether common risk SNPs cluster in discrete molecular pathways; pathway analyses based on current data have been equivocal, although providing some support for involvement of cell adhesion pathways. A related question is whether risk pathways capture discrete risk subgroups of patients defined by symptoms, clinical disorder, or some broader liability to developmental disorder? From these data it will also be possible to estimate whether a liability threshold of common variants is sufficient to cause schizophrenia, and whether this can be applied to individual risk prediction. At present, the lesson emerging from other disorders is that, where identified risk variants explain only a small proportion of total heritability (as is the case now for most diseases), or where heritability is modest, common risk variants are unlikely to have the discriminatory power to improve risk prediction [16].

## Structural genomic variation and schizophrenia

There is accumulating evidence for involvement of rare, genomic structural variation in schizophrenia. The two most persuasive schizophrenia genetics findings from the pre-genome era emerged from cytogenetic studies. In the first, a balanced translocation between chromosomes 1 and 11, causing a mutation of the gene disrupted-in-schizophrenia 1 (*DISC1*), strongly segregates with mental disorder in a large Scottish kindred [17]. Carrying the mutation has a large effect on liability to both schizophrenia and mood disorder. Outside this family, there is some evidence that other variants at the *DISC1 *locus are associated with other neuropsychiatric and cognitive phenotypes, but the translocation has not been identified in other families. The second finding is the association of schizophrenia with 22q11.2 deletion syndrome (22q11.2DS; also known as velo-cardio-facial syndrome), which has an incidence of 1 in approximately 4,000 live births and leads to a varied set of symptoms, including physical defects and learning disabilities. Phenotypic expression of 22q11.2DS is highly variable and can affect multiple organs and tissues, but carriers also have a 30-fold increased risk of schizophrenia. Animal models have been highly informative in clarifying how these mutations impact on brain development and function (reviewed in [18,19]). These models represent rare genetic forms, however, and it remains a matter of debate whether this limits their construct validity as models of schizophrenia [4].

The past five years have seen increased awareness of the presence and importance of submicroscopic deletions, duplications and rearrangements in the human genome. A seminal paper by Walsh and colleagues [20] identified an increased rate of novel deletions and duplications of genes in schizophrenia cases, particularly those with an early age at onset. Following on from the Walsh paper, two large consortia studies identified an association of copy number change at chromosome 1q21.1 and deletions of chromosome 15q13.3 with schizophrenia [21,22]. Subsequent studies have reported evidence for an association between schizophrenia and more copy number variations (CNVs), including both chromosomal microdeletions and microduplications (reviewed in [23,24]). Some of these span many genes, but the 2p16.3, 7q36 and 16p13.2 loci specifically implicate individual genes (*NRXN1*, which encodes a synaptic adhesion protein, *VIPR2*, which encodes a neuropeptide receptor, and *C16orf72*, respectively) [23-25]. Each of these loci is reported to increase schizophrenia risk from two- to ten-fold, making these interesting targets for further research [26-28] (Table 1).

Although individually rare, cumulatively the structural mutations identified to date involve approximately 5% of cases of schizophrenia. An unexpected finding is that these CNVs also confer risk for a range of other developmental phenotypes, including autism, learning disability, ADHD, seizure disorder, other physical anomalies and obesity. As an example, carriers of the 15q13.3 deletion have an increased rate of schizophrenia (6 to 9%), autism (approximately 10%), learning disability (approximately 50%) and epilepsy (approximately 30%), but a subset have no discernable clinical findings [29]. For each of these loci further studies are required to identify whether there are core features associated with the mutation (as for 22q11.2DS) or whether they involve such a wide range of phenotypic effects that syndromal classification will be difficult. Significantly, this phenotype list does not include bipolar disorder, where evidence for involvement of structural variation is more equivocal. However, this may reflect sample ascertainment as recent data suggest that CNVs contribute to the risk of early onset bipolar disorder (Jonathan Sebat, personal communication).

How much of schizophrenia risk involves rare mutations and how many of these require a background of other mutations or common risk effects for disease expression? These critical questions will define the rate of progress in translating genetic findings into biological insights. Although of much larger effect than the common variants defined as risk alleles by GWAS studies, most of the CNVs reported to date occur, albeit at lower frequency, in unselected control populations. In parallel with developing model systems for these mutations, it will be necessary to assess their penetrance and to establish whether more subtle phenotypes (for example, dyslexia or anxiety disorders) occur in seemingly unaffected individuals. Carefully defined control populations are important: the DISC1 family provide a salutory lesson as the original proband had a diagnosis of conduct disorder, rather than a major mental illness, and would have met control rather than case criteria in the standard case-control association study design. This highlights the complexity of the task at hand: it may be necessary to re-evaluate study design - and the results of previous studies - on the basis of new genetic information.

## Studying sequence-level mutations in schizophrenia

Exomic and whole genome sequence data will become available for hundreds, if not thousands, of schizophrenia patients in the next couple of years. What will this teach us? The limited reported sequence data currently available suggest that there may be an increased rate of potentially deleterious *de novo *mutations in schizophrenia patients compared to control subjects [30]. For example, an excess of missense variants has been reported in the gene *GRIN2B*, encoding the NMDA receptor subunit NR2B, in schizophrenia and autism but also with other neurodevelopmental phenotypes [31,32]. Assessing the significance of rare or unique mutations across the genome to disease manifestation, particularly if these fail to converge on the same genes, will be difficult as there may be just too many mutations to identify which have a causal role. One obvious starting point will be to assess sequence data at genes implicated by existing GWAS (for example, *TCF4*) and structural variation studies (for example, *NRXN1*). As the number of risk loci expands it will become possible to test specific hypotheses based on implicated risk pathways, although the success of this approach will require better pathway annotation. Lessons may also be learned from severe neurodevelopmental disorders where null mutations may have profound phenotypic effects on brain structure but less deleterious mutation may result in more subtle phenotypes, which could include schizophrenia [33]. As an example, Pitt-Hopkins syndrome, a developmental disorder with severe learning disability, can be caused by haploinsuffiency of either of two known schizophrenia risk genes, *TCF4 *or *NRXN1*. It is still too early to know whether such examples are representative, but based on the structural variation data it seems reasonable to investigate genetic mutations based on data generated across a range of neurodevelopmental phenotypes.

## From genes to biology

This is an interim phase in our understanding of the genetic architecture of schizophrenia. Conceptually the framework involves hundreds or even thousands of very modest risk alleles but also some number of rare mutations with a much larger effect on risk for certain individuals. Having rare, high penetrance mutations is a significant breakthrough, as it makes possible the development of model systems based on biology rather than on clinical symptomatology. This is particularly applicable where individual genes or point mutations are involved. Affected individuals become obvious targets for studies ranging from clinical investigation of their symptomatology, treatment response and outcome to imaging of their neural circuits and studies of blood cells reprogrammed as stem cells and differentiated as neurons in culture. In parallel, the mutations themselves can be modeled in cellular or animal systems. Increasingly sophisticated methods for examining neural circuits *in vivo *using viral tracing or optogenetics are also becoming available, but are beyond the scope of this article (reviewed in [34]).

For each implicated mutation it will be important to know the resultant cellular and behavioral phenotypes, the signaling or other mechanisms that result in these phenotypes and whether the phenotypes can be rescued by intervention with novel or known therapeutic agents. Taking the example of DISC1, we know that the normal regulation of neural progenitor proliferation by modulation of GSK3beta/beta-catenin signaling is disrupted in DISC1 mutants and it will be interesting to see if the same process is disturbed by other mutations. Experiments across mutations may define whether future therapies target a molecular risk mechanism common to most schizophrenia patients, a strategy that recent experiments on neuronal cell cultures derived from four unrelated schizophrenia patients suggests may be successful [35], or are much more focused on smaller groups based on many molecular etiologies. In turn, this raises a more profound question: are we investigating the clinical phenotype 'schizophrenia' or is this only one phenotypic outcome of different neurodevelopmental pathologies?

Answering this question will shape the future of nosology and define how psychiatric care is delivered in the future. Currently, DSM-IV classification draws a clear distinction between schizophrenia and other neurodevelopmental disorders. From analysis of genomic structural variation this seems artificial, as a significant subset of schizophrenia patients share overlapping molecular pathology with patients diagnosed with other developmental phenotypes, including learning disability, autism and epilepsy. Will future care to these families be delivered along existing guidelines based on clinical expertise or defined by molecular etiology?

Risk mutations may arise *de novo*, but may also be inherited, as is the case for 75% of individuals with 15q13.3 microdeletions. Within a family, carrying a risk mutation may represent risk for a constellation of developmental phenotypes. Systematic and standardized assessment of mutation carriers will be required to identify whether there are core features for specific genomic syndromes, to develop screening criteria to identify carriers and to define who should be screened. Although the known CNVs increase risk for a range of adverse outcomes, most CNVs are not 'causative' in a deterministic Mendelian genetics sense and their role in increasing risk is likely to be dependent on other genetic or environmental factors. To provide genetic counseling to families, a better model of the molecular framework that underlies these phenotypes will be required. Current estimates of the penetrance of known risk mutations are based on ascertainment from highly selected populations (for example, patients with developmental disorders) who may have a higher burden of other mutations than is representative in the general population. For the molecular data to be meaningful, prospective studies to group genetic risk factors and collect information on environmental risk factors will be required. For 'schizophrenia' this may be challenging as the known environmental risk factors are typically small (for example, obstetric complications), difficult to quantify (for example, cannabis exposure), or difficult to interpret (for example, urban living).

Returning to patients A and B, enrolled as cases in a 'schizophrenia' research study, this may have significant ramifications for future schizophrenia studies as we try to understand differences in symptoms, treatment response, course of illness and outcome evident in clinical populations. Recognizing that a proportion of patients carry high penetrance risk mutations may demarcate a 'syndromal' form of schizophrenia, or patients at risk of neurodevelopmental phenotypes including psychotic symptoms, much as is happening within autism spectrum disorders now. Within this 'syndromal schizophrenia' group, further distinctions may be possible based on molecular etiology. A corollary of identifying patients with more genetic forms of a disorder is that this might also identify patients with a less genetic form. It is too early to tell whether having different levels of molecular risk is associated with differences in symptom severity, treatment response or outcome. But this may emerge as useful information in advising patients on risk of recurrence, relapse prevention or therapeutics. Having this information may also influence how doctors interpret symptoms: in a carrier of a 15q13.3 deletion, are insidious negative symptoms due to schizophrenia, reflective of social impairments due to an autistic spectrum disorder, or actually just a feature of the neural systems affected? In the real world, clinicians know that many patients do not fit neatly within existing diagnostic categories. As an illustrative example, a patient may have attended psychiatric services since childhood and been diagnosed with developmental delay, 'behavioral problems', and subsequently an autistic spectrum disorder, and in adulthood schizophrenia. Knowing that this patient has a pathogenic *NRXN1 *mutation may provide a much sounder basis on which to diagnose or prescribe treatment.

Projecting (speculatively) to the clinic of 2025, it may be that for patient A, after the molecular diseases that cause schizophrenia are excluded, only a modest burden of common risk variants are identified. The episode is identified as proximal to a psychological stressor; she receives a focused psychotherapy to address how she dealt with the stressor, the risk of recurrence is low and pharmacotherapy is not indicated. Patient B is identified as having a mutation that has a functional effect on a signaling mechanism, this is known to respond to an existing therapy, and his medication is altered appropriately. He also has a significant family history of seizure disorder and autism and the family is being investigated further by clinical geneticists to further our understanding of the genetic basis of, and relationships amongst, these conditions.
